# Comparison of Neural Network Structures for Identifying Shockable Rhythm During Cardiopulmonary Resuscitation

**DOI:** 10.3390/jcm14030738

**Published:** 2025-01-23

**Authors:** Sukyo Lee, Sumin Jung, Sejoong Ahn, Hanjin Cho, Sungwoo Moon, Jong-Hak Park

**Affiliations:** 1 Department of Emergency Medicine, Korea University Ansan Hospital, Ansan-si 15355, Republic of Korea; sukyolee@korea.ac.kr (S.L.); sejoongahn@naver.com (S.A.); chohj327@gmail.com (H.C.); yg9912@korea.ac.kr (S.M.); 2 Core Research & Development Center, Korea University Ansan Hospital, Ansan-si 15355, Republic of Korea; sumini1019@korea.ac.kr

**Keywords:** cardiopulmonary resuscitation, electrocardiogram, deep learning

## Abstract

**Background/Objectives**: Minimizing interruptions in chest compressions is very important when resuscitating patients with cardiac arrest. Recently, research has analyzed electrocardiograms (ECGs) during chest compressions using convolutional neural networks (CNNs). This study aimed to compare the accuracy of deeper neural networks and more advanced structures. **Methods**: ECGs with chest compression artifacts were obtained from patients who visited the emergency department of Korea University Ansan Hospital from September 2019 to February 2024. We compared the accuracy of a deeper CNN, long short-term memory (LSTM), and a CNN with an attention mechanism and residual block against a reference model. The input of the model was 5 s ECG segments with compression artifacts, and the output was the probability that the ECG with the artifacts was a shockable rhythm. **Results**: A total of 1889 ECGs with compression artifacts from 172 patients were included in this study. There were 969 ECGs annotated as shockable and 920 as non-shockable. The area under the receiver operating characteristic curve (AUROC) of the reference model was 0.8672. The AUROCs of the deeper CNN for five and seven layers were 0.7374 and 0.6950, respectively. The AUROCs of LSTM and bidirectional LSTM were 0.7719 and 0.8287, respectively. The AUROC of the CNN with the attention mechanism and residual block was 0.7759. **Conclusions**: CNNs with deeper layers or those incorporating attention mechanisms, residual blocks, and LSTM architectures did not exhibit better accuracy. To improve the model accuracy, a larger dataset or advanced augmentation techniques may be required, rather than complicating the structure of the model.

## 1. Introduction

When resuscitating a patient with cardiac arrest, it is very important to follow the chain of survival [1,2,3]. The chain of survival includes early recognition, high-quality cardiopulmonary resuscitation (CPR), and rapid defibrillation. High-quality CPR is also required both before and after defibrillation, and interruptions to CPR should be minimized [4,5,6,7,8]. However, CPR is unavoidably interrupted every 2 min to analyze the electrocardiogram (ECG) rhythm. If CPR could continue uninterrupted while analyzing the ECG, more effective chest compressions could be delivered, potentially improving patient outcomes.

Various methods have been investigated to analyze ECGs during CPR to minimize interruptions. Techniques have been proposed to eliminate artifacts using adaptive filters or thoracic impedance measurements [9]. Advances in artificial intelligence (AI) have led to the development of machine learning methods that can more accurately classify ECGs with artifacts [10,11]. Recently, shockable rhythm analysis during CPR has been studied using deep learning methodologies, demonstrating acceptable performance [12,13,14,15]. The literature provides a detailed review of the characteristics of several machine learning and deep learning methodologies for ECG analysis during chest compression [16]. Utilizing thoracic impedance in deep learning methodologies to improve performance has also been proposed, but the accuracy decreased, and it was reported that adding a recurrent layer improved the sensitivity by about 1% [17]. Most studies have used prehospital automated external defibrillator (AED)-acquired or artificially synthesized ECGs, utilizing a convolutional neural network (CNN). However, few studies have been conducted using in-hospital data.

Several studies have analyzed clean ECGs to classify arrhythmias and diagnose acute myocardial infarction [18,19,20,21]. CNNs are reported to be the most frequently used neural network structures for analyzing ECGs. Recently, several studies have applied recurrent neural networks and long short-term memory (LSTM) models to improve the performance. Hybrid structures combining CNNs with other architectures have also been explored. In 2017, the transformer architecture and attention mechanisms were developed for language processing and began to be utilized in the medical field [22,23]. Subsequently, studies have been conducted to improve the ECG analysis performance by incorporating attention mechanisms [24,25,26]. However, despite the evolution of deep learning techniques and the use of state-of-the-art methods for ECG analysis, CNNs remain the predominant approach for analyzing ECGs during CPR.

Recently, our research team reported a neural network model that used a CNN to predict shockable rhythms from ECGs with CPR artifacts acquired in-hospital [25]. In this study, we hypothesized that more advanced deep learning methodologies, including deeper CNNs, LSTM, and CNNs with attention mechanisms and residual blocks, would result in higher accuracy.

## 2. Materials and Methods

### 2.1. Data Source and Clinical Setting

A continuous bio-signal database of data collected in the emergency department of Korea University Ansan Hospital from September 2019 to February 2024 was used as the data source. Patients who arrived at the ED with an out-of-hospital cardiac arrest (OHCA) and those who had an in-hospital cardiac arrest (IHCA) during their ED stay were screened for enrollment from the database. Patients who achieved a return of spontaneous circulation (ROSC) before arriving at the ED, those for whom resuscitation was not initiated, or those aged < 18 years were excluded. Furthermore, patients who did not enter the resuscitation room, those who experienced IHCA outside the resuscitation room, and those with no recorded continuous vital sign data were excluded. A total of 1889 5 s single-lead ECGs (Lead II) were extracted from 172 patients with cardiac arrest. Of these, 969 ECG segments had shockable rhythms, and 920 ECG segments had non-shockable rhythms. Detailed characteristics and the ECG annotations of the patients included in this study are presented in Table 1. The characteristics of the patients and electrocardiograms have also been described in previous studies [27].

Korea University Ansan Hospital is a tertiary teaching hospital with a level 1 ED, which has approximately 48,000 patient visits annually. Our institution prospectively collects continuous bio-signals from critically ill patients visiting the ED for patient safety and quality improvement. The database consists of continuous ECG waveforms, plethysmography, invasive arteriography, capnography, and numerical vital signs. The patient monitoring system used was a GE CARESCAPE B850 (GE Healthcare, Wilmington, DE, USA). Continuous bio-signal data were captured by connecting the monitor to a laptop using VitalRecorder software (version: 1.12.1). VitalRecorder, an open-source software package, was designed to record signals from various monitors [28,29].

### 2.2. Ethical Statement

This study adhered to the principles of the Declaration of Helsinki. Our institution waives the need for informed consent for the prospective collection of predefined variables for patient safety and quality improvement purposes. Therefore, the need for consent for prospective data collection from a continuous bio-signal database was waived. The study was approved by the Institutional Review Board (IRB) of Korea University Ansan Hospital (IRB number: 2024AS0028; date of approval: 16 January 2024), which waived the requirement for informed consent due to the retrospective nature of the EMR review and bio-signal data matching. Additional IRB approval was obtained for a performance comparison study based on the advanced model structures (IRB number: 2024AS0190; date of approval: 30 July 2024). The requirement for informed consent was waived owing to the retrospective nature of the study.

### 2.3. Data Processing

**Figure 1:**
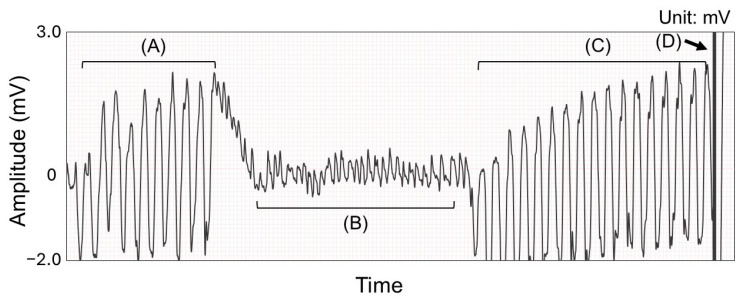
Data processing and labeling of electrocardiogram (ECG) of shockable rhythm. (**A**) ECG with cardiopulmonary resuscitation (CPR) artifact before rhythm analysis. (**B**) ECG without CPR artifact. This portion was used for labeling. (**C**) ECG with CPR artifact after rhythm analysis during preparation of defibrillation. (**D**) Moment of defibrillation. (**A**,**C**) were used for neural network training and validation.

**Figure 2:**
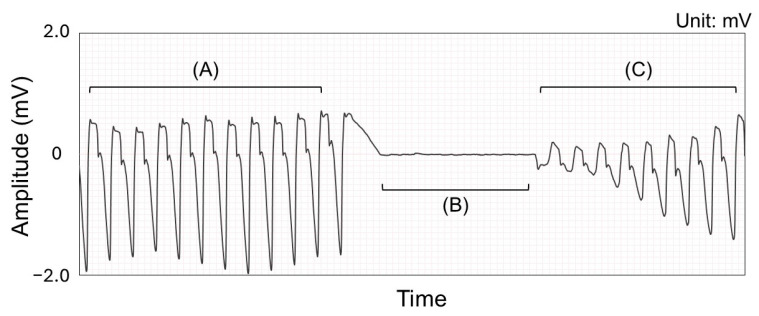
Data processing and labeling of electrocardiogram (ECG) of non-shockable rhythm. (**A**) ECG with cardiopulmonary resuscitation (CPR) artifact before rhythm analysis. (**B**) ECG without CPR artifact. This portion was used for labeling. (**C**) ECG with CPR artifact after rhythm analysis. (**A**) was used for neural network training and validation.

We extracted 5 s ECG segments with compression artifacts immediately before chest compression interruption in each resuscitation cycle (Figure 1 and Figure 2). Five 5 s ECGs were extracted at 1 s intervals with 4 s of overlap per cycle in the case of shockable rhythms. This overlapping approach was used to mitigate class imbalance issues for shockable rhythms. ECGs with compression artifacts during the preparation of defibrillators were extracted at 5 s intervals without an overlap to prevent redundancy and ensure data diversity. In the case of non-shockable rhythms, a single ECG was extracted immediately before compression interruption.

The ECGs were annotated by three board-certified emergency medicine physicians. Annotation was performed using an ECG recorded from the moment chest compressions were stopped, and the ECG was free of artifacts. Three physicians independently performed the annotations. Annotations that were unanimously agreed upon by the three physicians were used, and any disagreements were resolved through discussion.

The original ECG signals were recorded at a frequency of 300 Hz. To improve the computational efficiency and reduce high-frequency noise, we downsampled the signals to 100 Hz. We checked for missing values in the ECG data, and any ECG segments containing missing values were excluded to maintain the data quality. Additionally, the ECG data timestamps, originally in Unix time, were converted to Korean Standard Time to accurately align the ECG signals with clinical events.

In this study, the ECG data were assigned for training, validation, and testing on a per-patient basis. This was because a simple proportional allocation (such as a 7:2:1 ratio) would result in one patient’s ECG being assigned to every set. This would mean that ECGs similar to those already learned in the training set would be present in the test set, potentially causing the overestimation of performance. However, when assigned by the patient, the number of ECGs acquired for each patient varied; therefore, the training, validation, and test ratios for each model were different. To address the class imbalance, we used random oversampling for classes with small amounts of data. We performed patient-wise data splitting to ensure that data from the same patient did not appear in multiple sets, thereby preventing data leakage. In the training set, the classes were balanced by randomly duplicating samples from the minority class until both classes had equal representation. This approach maintained the integrity of the original ECG signals without introducing synthetic artifacts.

Because all ECG segments from the same patient had to reside in the same subset (training, validation, or test), each advanced model ended up with slightly different numbers of training, validation, and test samples after random splitting. Moreover, model-specific hyperparameter tuning and class-balancing strategies (e.g., oversampling, augmentation) further contributed to minor variations in the final split sizes. Table 2 provides detailed information on the data splits used for each advanced model.

### 2.4. Architecture of Neural Network and Training

#### 2.4.1. Reference Model [27]

The reference model used in this study was a foundational one-dimensional CNN designed to analyze ECG data containing compression artifacts incurred during CPR. The model processes the input signal through an initial convolutional layer, which extracts critical features using batch normalization and rectified linear unit (ReLU) activation functions to enhance the learning stability and mitigate overfitting. Three convolutional layers, each followed by max pooling layers, efficiently distill the signals into significant features. A fully connected layer subsequently computes class probabilities based on the extracted features. The streamlined architecture of the reference model delivered a reliable performance and served as a benchmark for evaluating the efficacy of more advanced models.

#### 2.4.2. CNN Model with Deeper Layers

A CNN model with deeper layers extends the basic architecture to capture the complex patterns inherent in the ECG data. This model incorporates either five or seven convolutional layers, with each layer incrementally increasing the number of output channels, thereby enabling the extraction of deep features. The use of batch normalization and ReLU activation across the layers promotes stable convergence and robustness. This architecture excels in scenarios involving high-complexity data, thereby facilitating the recognition of intricate ECG patterns. However, regularization techniques, such as dropout, are essential to prevent overfitting because of the increased depth of the model.

#### 2.4.3. LSTM Model

The LSTM model, optimized for sequential data processing, effectively captured temporal patterns within the ECG data. Each LSTM layer retains hidden states, ensuring the continuity of critical information throughout the input sequence. By stacking multiple LSTM layers, the model learns complex temporal dependencies, and the final output is determined via a fully connected layer. Bidirectional LSTM (Bi-LSTM) processes data in both the forward and backward directions, enriching the contextual understanding, which is particularly advantageous for analyzing the temporal dependencies of ECG signals. Bi-LSTM’s capabilities enhance the model’s proficiency in comprehending and predicting dynamic cardiac rhythms. In addition to basic Bi-LSTM, this study incorporated an advanced version, the advanced Bi-LSTM model. This model extends the basic Bi-LSTM architecture by including an additional fully connected layer after the Bi-LSTM output. The model first extracts temporal features using Bi-LSTM and then refines the learned features through an additional fully connected layer, followed by dropout to prevent overfitting. This additional layer facilitates a more refined feature representation, ultimately leading to improved classification performance. The enhanced advanced Bi-LSTM model is particularly suitable for more complex ECG signal patterns, offering greater flexibility in learning from the data while maintaining robustness against overfitting through regularization techniques, such as dropout.

#### 2.4.4. CNN Model with Residual and Attention Mechanisms

The CNN model incorporating residual and attention mechanisms builds on the architecture of the reference model described in Section 2.4.1. It was engineered to minimize information loss and emphasize the salient features within the data. Residual connections facilitate effective learning in deep networks by alleviating the vanishing gradient problem and ensuring a seamless information flow through direct links between the input and output signals. The self-attention mechanism further empowers the model to concentrate on pivotal features, accentuating crucial signal segments. This architecture excels in extracting meaningful information from complex data, thereby improving the accuracy of ECG analysis. The synergy of the residual and attention mechanisms enhances the model interpretability and augments its practical utility in clinical settings.

#### 2.4.5. Overview of Model Architecture

In addition to the reference model described in Section 2.4.1, this study investigated several advanced architectures, including deeper CNNs, LSTM-based models, and CNNs with residual and attention blocks. Table 3 summarizes the core architectural components of each model, highlighting the number of convolution or LSTM layers, the presence of residual or attention modules, and key parameters such as the hidden size or dropout rate. The 5 s ECGs with compression artifacts were input as 1 × 500 data points and passed through the neural networks. The final output was the probability that the input ECG would exhibit a shockable rhythm. The output layer of all models utilized a sigmoid activation function for binary classification (shockable vs. non-shockable).

Hyperparameter tuning was performed using a grid search and cross-validation to evaluate different combinations of parameters, such as the learning rate and batch size. Specifically, we performed a grid search combined with five-fold cross-validation to systematically evaluate the hyperparameters. We tested learning rates ranging from 1 × 10^−5^ to 1 × 10^−3^ and batch sizes of 64, 128, and 256. Regularization techniques, such as dropout rates between 0.3 and 0.5, were applied to prevent overfitting. The optimal hyperparameter combination was selected based on the performance metrics from the validation dataset.

#### 2.4.6. Loss Function

To optimize the performance of the proposed models, we used the binary cross-entropy (BCE) loss function, which is widely adopted for binary classification tasks. The BCE loss is mathematically defined in Equation (1).

(1) $$L=-\frac{1}{N}\sum_{i=1}^{N}\left[y_{i}\cdot \log\left(\hat{p_{i}}\right)+\left(1-y_{i}\right)\cdot \log\left(1-\hat{p_{i}}\right)\right]$$

Equation (1). Definition of the binary cross-entropy (BCE) loss function.

In Equation (1), N represents the total number of samples, $y_{i}\in \{0,1\}$ is the true label for the ith sample, and ${\hat{p}}_{i}$ is the predicted probability that the sample belongs to the positive class (i.e., a shockable rhythm). The BCE loss penalizes incorrect predictions by assigning higher penalties to the terms corresponding to the true class label. This ensures that the model minimizes classification errors for both shockable and non-shockable rhythms during training.

The Adam optimizer was employed to minimize the BCE loss, with hyperparameters (e.g., the learning rate) tuned empirically to ensure convergence. The BCE loss is particularly suitable for imbalanced datasets, as it inherently adjusts the loss contribution from each class based on their predicted probabilities. This is a crucial advantage given the inherent imbalance in the shockable versus non-shockable rhythm distributions in our dataset.

#### 2.4.7. Model Ensemble and Decision Process

We trained five models for each architecture with random patient assignments and then ensembled these five models. For ensemble prediction, we used probability averaging, in which the predicted probabilities from each of the five models were averaged to produce the final prediction. A rhythm was considered shockable if the arithmetic average of the probabilities given by each of the five models was greater than 0.5, and vice versa for non-shockable rhythms. This method helped mitigate individual model biases and reduced variance, leading to more stable and reliable performance. The best performing ensemble model was selected from the multiple ensemble models.

### 2.5. Statistical Analysis

The performances of the different models according to the patient assignment were presented as the accuracy, area under the receiver operating characteristic curve (AUROC), area under the precision–recall curve, sensitivity, specificity, positive predictive value, and negative predictive value. We compared the accuracy of each model advancement method using the results of a previous study with the same dataset as a reference [27]. Python 3.1.1 and PyTorch 2.2 were used for the model training and statistical analysis. We used four NVIDIA GeForce RTX 4090 graphics processing units in parallel for the model training and statistical analysis.

## 3. Results

### 3.1. Performance of CNN with Deeper Convolutional Layers

Table 4 lists the performance metrics according to the number of convolutional layers in the CNN model. As the number of convolutional layers increased gradually from three to seven, the AUROC decreased progressively. While the specificity increased slightly, the sensitivity dropped sharply. The accuracy also exhibited a gradual decline.

### 3.2. Performance of LSTM and Bi-LSTM Models

Table 5 presents the performance metrics for the LSTM and Bi-LSTM models. The LSTM and advanced Bi-LSTM models demonstrated slightly higher specificity than that of the reference model; however, all other performance metrics were lower than those of the reference model. Compared with that of the reference model, the performance of the Bi-LSTM model was similar in terms of the AUROC, area under the precision–recall curve, and accuracy. The sensitivity was higher for the reference model, whereas the specificity was higher for the Bi-LSTM model.

### 3.3. Performance of CNN with Attention Mechanism and Residual Block

Table 6 outlines the performance metrics of the CNN model integrated with an attention mechanism and residual blocks. When the residual block and attention mechanism were added to the baseline CNN model, the performance did not improve. The AUROC, sensitivity, specificity, and accuracy decreased in the model with residual blocks and attention mechanisms.

## 4. Discussion

The ability to analyze ECGs while performing CPR is expected to improve patient outcomes. Indeed, the DEFI 2022 study showed that using the algorithm for analyzing the cardiac rhythm while performing chest compression (AWC) achieved a higher chest compression fraction, as well as a survival benefit for early intervention in OHCA in public places [30]. An AED with cprINSIGHT also reduced the pre-shock pause and significantly increased the chest compression fraction [31]. AWC and cprINSIGHT analyzed rhythms during compression without requiring confirmation from a clean ECG in 72.3% and 70.4% of cases, respectively.

If the accuracy of analyzing an ECG during CPR is as accurate as that of analyzing a clean ECG, a better clinical prognosis could be expected. In our previous work, we analyzed ECGs during CPR with a deep learning methodology using convolutional neural networks [27]. However, the accuracy of the model was much lower than that of a conventional AED on a clean ECG. We therefore experimented with different structures of neural networks to improve the performance.

We adopted an increased depth of convolutional layers, introduced LSTM, and added a residual block and an attention mechanism to improve the performance of a neural network that analyzes ECGs during CPR and identifies shockable rhythms for patients with cardiac arrest. However, all the methodologies adopted in this study had a similar or worse accuracy than that of the reference model.

This is the first study to analyze ECGs during CPR with LSTM and attention mechanisms using data from real in-hospital patients. It is also the first study to compare the performances of CNNs, LSTM, and attention mechanisms identifying shockable rhythms using deep learning.

In general, CNNs are known for their ability to recognize and analyze images or patterns and require relatively little computational power. This is the most popular methodology in ECG studies that use deep learning [19,20]. LSTM models are strong at analyzing time series data but require relatively more computational power. Recently, an increasing number of studies have used LSTM to analyze ECGs [19,20]. Because ECGs are time series data and the pattern recognition of ECGs is also important, it is critical to use CNNs and LSTM appropriately depending on the purpose.

In our study, we observed a decrease in accuracy as we increased the number of convolutional layers from three. This finding is consistent with those of previous studies. Isasi et al. used 3319 prehospital-acquired ECGs, showing the best performance when using three convolutional layers and overfitting with decreasing accuracy when using more than four layers [14]. Jekova et al. used approximately 11,000 prehospital-acquired ECGs and found optimal accuracy with three convolutional layers, with a significant decrease in accuracy for more than five layers [15]. Conversely, Hajeb-M et al. created 38,016 “CPR-contaminated” ECGs by synthesizing ECGs without chest compressions and adding compression artifacts to asystoles [17]. When approximately 40,000 artificially synthesized ECGs were used, the best performance was achieved with seven CNN layers. Despite the fact that validation was also performed on the artificially synthesized ECGs, there are questions about whether this can be applied to real patients. They found that deeper CNN layers performed better by using a larger amount of data.

The decrease in performance observed with deeper CNN models may be due to overfitting. As the complexity of the model increases, more data are required to generalize well. Our dataset, which consisted of 1D ECG signals with relatively simple patterns and a limited sample size, may not provide sufficient information for such complex models to learn effectively. The deeper models may have overfitted the training data, capturing noise rather than meaningful patterns, which led to reduced performance on the validation and test sets. Therefore, focusing on collecting more data or applying advanced augmentation techniques might be more effective than complicating the model structure to improve accuracy. We need to acquire more training data for higher accuracy by collecting data from multiple institutions. In addition, artifacts differ depending on the environment; therefore, artifacts should be collected from various environments, including both hospitals and prehospital settings [27,32].

The accuracy did not improve when ECGs with artifacts were analyzed using LSTM. Using Bi-LSTM resulted in a higher AUROC than that of the other LSTM methodologies but lower accuracy than that of the reference model. In contrast, the specificity was higher in the Bi-LSTM model than in the reference model, and the sensitivity was higher in the reference model. Both the sensitivity and specificity were reduced when the attention mechanism and residual block were used. Considering this, there may be a synergetic effect when combining CNNs and Bi-LSTM; therefore, further research is required.

The computational power of the ECG analysis device in both the training and inference phases must be considered when building a model. Defibrillators must provide real-time predictions by analyzing ECGs to rapidly deliver shocks while performing chest compressions. In one study analyzing ECGs using a CNN model, it took 0.383 s for the AED to analyze 7 s of ECGs [33]. In general, LSTM architectures require more computations than CNNs. In some cases, it may not be possible to execute the LSTM model in an AED. If an AED requires more than a few seconds to make an inference, it would be very difficult to use in clinical practice. Therefore, if more complex models are used to increase the performance, the AED will require a more powerful processor. Alternatively, we should find a way to achieve better performance using a simpler model that does not require significant computation. The model’s compression can also be optimized through frameworks, such as TensorRT or ONNX Runtime.

A major challenge in AI model development is the lack of data bias. Traditional data augmentation techniques have been employed to address this issue; however, their effectiveness has been limited. Multicenter acquisition to collect a large amount of data is essential. In addition, artificially synthesized ECGs may be considered to improve the accuracy. Recently, generative AI using diffusion techniques for data augmentation has been actively used [34,35,36]. However, there is no guarantee that synthesized ECGs can completely replace ECGs from real patients; therefore, at least validation should be performed on data from real patients.

This study has several limitations. All ECGs used in this study were obtained during cardiac arrest. The ECGs with ventricular tachycardia (VT) included in this study were pulseless. All ECGs with pulseless electrical activity were clearly identified as pulseless by the physicians. The most important limitation of this study was that we could not identify how the model would respond to VT rhythms with pulses and sinus rhythms with the return of spontaneous circulation. In addition, VT and ventricular fibrillation, which are shockable rhythms, have different ECG morphologies. Categorizing these two rhythms into a single group, called a shockable rhythm, has the potential to decrease the performance of the model. In future studies, a model should be developed to categorize ventricular fibrillation, VT, pulseless electrical activity, and asystoles. This would allow physicians to manually check the pulse, if necessary. Another limitation is that we were unable to determine the statistical significance when comparing the performance of each model. To determine whether the difference in an AUROC is statistically significant, bootstrapping is required to determine the confidence intervals and test for statistical significance, which was not feasible because of computational power limitations. Finally, this study used a single-center, retrospective, observational design. We divided the patients into training, validation, and test sets but did not perform external validation with other institutional data. Bias due to the research design cannot be excluded. In this regard, we are currently building a research consortium and collecting data from multiple hospitals; therefore, we plan to conduct external validation as a follow-up study.

## 5. Conclusions

CNNs with deeper layers or those with attention mechanisms, residual blocks, and LSTM architectures did not exhibit better accuracy. This may be due to the low data complexity of ECGs with artifacts or the small dataset. To increase the accuracy of the model, a larger number of datasets or advanced augmentation techniques are required, rather than complicating the model structure. A more accurate model could result in a higher chest compression fraction and better survival outcome.

## Figures and Tables

**Table 1:** The characteristics of the patients included in the analysis and the annotation of electrocardiograms.

Variable	Numbers
Patient information	
The number of patients	172
Sex, male, *n* (%)	118 (16.6%)
Age, median (IQR)	66 (54–81)
Recorded resuscitation cycle per patient, median (IQR)	5 (2–8)
Electrocardiogram annotation, *n* (%)	
Shockable rhythm	969 (51.3%)
Ventricular fibrillation	822 (43.5%)
Pulseless ventricular tachycardia	147 (7.8%)
Non-shockable rhythm	920 (48.7%)
Asystole	359 (19.0%)
Pulseless electrical activity	561 (29.7%)

IQR: interquartile range.

**Table 2:** Summary of the data splits for each advanced model (reference model excluded).

Model	Total	Training	Validation	Test
CNN model (*n* = 5)	1889	1640	143	106
CNN model (*n* = 7)	1889	1548	190	151
LSTM model	1889	1488	246	155
Bi-LSTMmodel	1889	1446	206	237
Advanced Bi-LSTM model	1889	1569	172	148
CNN (*n* = 3) +Residual +Attention	1889	1573	154	162

CNN: convolutional neural network; LSTM: long short-term memory; Bi-LSTM: bidirectional long short-term memory.

**Table 3:** Overview of the six deep learning architectures investigated in this study (reference model excluded).

Model	Description	Architecture and Key Parameters
CNN model (*n* = 5)	1D CNN with 5 convolution blocks Suited for moderate data complexity	5 × (Conv → BN → ReLU → MaxPool) Flatten → Dropout (0.5) → FC (128→1) Output: Sigmoid
CNN model (*n* = 7)	1D CNN with 7 convolution blocks Deeper structure for more complex patterns	7 × (Conv → BN → ReLU → MaxPool) Flatten → Dropout (0.5) → FC (128→1) Output: Sigmoid
LSTM model	Unidirectional LSTM stacked in 5 layers Focuses on time series patterns in ECG data	LSTM (5 layers, hidden_size = 1024, batch_first) Last time step → FC (1) Output: Sigmoid
Bi-LSTM model	Bidirectional LSTM stacked in 5 layers Captures both forward and backward temporal context	Bi-LSTM (5 layers, hidden_size = 1024 × 2) Last time step → FC (1) Output: Sigmoid
Advanced Bi-LSTM model	Bi-LSTM plus extra FC layer and dropout Helps refine features and reduce overfitting	Bi-LSTM (5 layers, hidden_size = 1024 × 2) Last time step → FC (1024) → ReLU → Dropout → FC (1) Output: Sigmoid
CNN (*n* = 3) + Residual + Attention	1D CNN with 3 blocks Each block includes a residual connection and self-attention mechanism	3 × (ResBlock + SelfAttention + MaxPool) Flatten → Dropout (0.5) → FC (128→1) Output: Sigmoid

CNN: convolutional neural network; LSTM: long short-term memory; Bi-LSTM: bidirectional long short-term memory; Conv: convolutional layer; BN: batch normalization; FC: fully connected layer; ReLU: rectified linear unit.

**Table 4:** Performance comparison of deeper convolutional neural network models with the reference model.

Model	Test Cases	AUROC	Sensitivity	Specificity	AUPRC	Accuracy	PPV	NPV
Reference model (*n* = 3) [27]	227	0.8672	91.4%	61.0%	0.8695	74.9%	66.4%	89.3%
CNN model (*n* = 5)	106	0.7374	56.3%	80.7%	0.7515	69.6%	72.1%	69.2%
CNN model (*n* = 7)	151	0.6950	23.3%	84.5%	0.6717	52.1%	61.7%	49.5%

*n*: the number of convolutional layers; CNN: convolutional neural network; AUROC: area under the receiver operating characteristic curve; AUPRC: area under the precision–recall curve; PPV: positive predictive value; NPV: negative predictive value.

**Table 5:** Performance comparison of the long short-term memory (LSTM) and bidirectional LSTM (Bi-LSTM) models with the reference model.

Model	Test Cases	AUROC	Sensitivity	Specificity	AUPRC	Accuracy	PPV	NPV
Reference model [27]	227	0.8672	91.4%	61.0%	0.8695	74.9%	66.4%	89.3%
LSTM model	155	0.7719	66.1%	74.4%	0.6453	71.1%	63.9%	77.3%
Bi-LSTM model	237	0.8287	69.7%	80.0%	0.8011	74.3%	82.5%	69.3%
Advanced Bi-LSTM model	148	0.7236	66.7%	65.6%	0.6819	66.1%	69.3%	67.6%

LSTM: long short-term memory; AUROC: area under the receiver operating characteristic curve; AUPRC: area under the precision–recall curve; PPV: positive predictive value; NPV: negative predictive value.

**Table 6:** Performance comparison of convolutional neural network models with residual blocks and attention mechanisms against the reference model.

Model	Test Cases	AUROC	Sensitivity	Specificity	AUPRC	Accuracy	PPV	NPV
Reference Model [27]	227	0.8672	91.4%	61.0%	0.8695	74.9%	66.4%	89.3%
CNN (*n* = 3) +Residual +Attention	162	0.7759	78.5%	58.5%	0.6465	66.9%	59.4%	84.2%

*n*: the number of convolutional layers; CNN: convolutional neural network; AUROC: area under the receiver operating characteristic curve; AUPRC: area under the precision–recall curve; PPV: positive predictive value; NPV: negative predictive value.

## Data Availability

The datasets used and/or analyzed during the current study are available from the corresponding author on reasonable request.
